# Determination of Low Concentrations of Mercury Based on the Electrodeposition Time

**DOI:** 10.3390/nano14110981

**Published:** 2024-06-05

**Authors:** Kenshin Takemura, Wataru Iwasaki, Nobutomo Morita, Shinya Ohmagari, Yasunori Takaki, Hitomi Fukaura, Kazuya Kikunaga

**Affiliations:** 1 Sensing System Research Center, National Institute of Advanced Industrial Science and Technology (AIST), 807-1 Shuku-Machi, Tosu 841-0052, Saga, Japan; wataru.iwasaki@aist.go.jp (W.I.); morita.nobutomo@aist.go.jp (N.M.); shinya.ohmagari@aist.go.jp (S.O.); k-kikunaga@aist.go.jp (K.K.); 2 Sakamoto Lime Industry Co., Ltd., 273-1 Simo, Tamana 865-0013, Kumamoto, Japan; k-kaihatu01@sakamoto-lime.com (Y.T.); k-kaihatu02@sakamoto-lime.com (H.F.)

**Keywords:** time response, nanoparticle, electrochemical detection, mercury, environment

## Abstract

Soil plays a crucial role in human health through its impact on food and habitation. However, it often contains toxic heavy metals, with mercury being particularly hazardous when methylated. Currently, high-sensitivity, rapid detection of mercury is achievable only through electrochemical measurements. These measurements require pretreatment of the soil sample and the preparation of a calibration curve tailored to the sample’s condition. In this study, we developed a method to determine the environmental standard value of mercury content in soil by significantly reducing the pretreatment process. Our approach involves analyzing current peaks from electrodeposition times using specific electrodes and solvent settings. This method demonstrates low error rates under low concentration conditions and can detect mercury levels as low as 0.5 ppb in soil leachate and reagent dilution series. This research facilitates the determination of low mercury concentrations in solutions containing various soil micro-compounds without the need for calibration curves.

## 1. Introduction

Mercury can cause severe symptoms in humans even at low exposure levels. Consequently, it is strictly controlled as a toxic substance [1,2]. Once released into the environment, mercury can be bioaccumulated in the food chain through methylation by bacterial action [3,4]. This bioaccumulation results in concentrated mercury being ingested by humans, primarily through the consumption of fish [5,6,7]. Therefore, various statistical studies have been conducted on mercury levels in humans, with higher levels detected in the urine, blood, and hair of individuals who consume large amounts of fish or frequently excavate soil for gold mining [8].

Generally, mercury content in soil and groundwater is measured using advanced and highly sensitive equipment such as atomic absorption spectrometry [9,10,11]. However, the removal of mercury from contaminated sources is extremely costly [12]. These methods can detect mercury at concentrations as low as 0.5 ppb with an accuracy of 98% or higher. Nevertheless, their practical utility is limited due to the large amounts of reagents required and the extended sample preparation times, making them expensive for routine application. For anthropogenic sources of environmental pollution, simple and regular inspections can prevent large-scale contamination [13,14]. Therefore, a simple and sensitive detection system that can detect low mercury concentrations quickly is needed. The primary challenge in rapid inspection implementation is the extraction method [15]. Chemical and physical methods commonly minimize the effects of the soil matrix, requiring long extraction times of at least 6 h, which dominate the inspection process. Recently, biological detection methods using plants and microorganisms have been reported [16]. While suitable for discussing environmental risks, these methods are still difficult to quantify accurately [17]. To shorten the extraction process, a one-step pretreatment method using a sensor resistant to the effects of soluble foreign substances during inspection is needed, which would reduce inspection time [18,19,20].

Electrochemical mercury detection has been extensively studied as a potential simple method for measuring low concentrations in sample solutions [21]. Sensitivity enhancement technologies based on increased electron transfer rates and specific surface areas using nanomaterials are expected to be core technologies for next-generation electrochemical sensors [22,23,24]. However, contamination caused by the adsorption of foreign substances at the electrode interface, which is highly sensitive due to its high specific surface area, significantly affects the signal [25]. Utilizing the simplicity and high sensitivity of electrochemical methods in contamination-resistant conditions could lead to an unprecedentedly simple inspection method.

**Figure 1:**
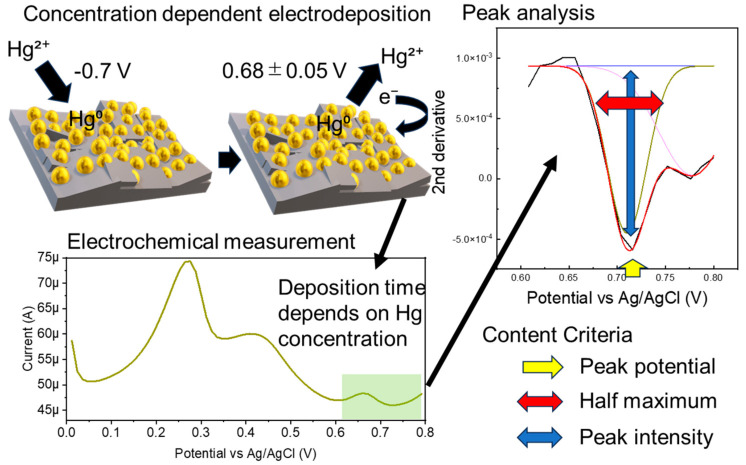
Schematic of the electrochemical measurement and analysis procedure used to determine the environmental standard of mercury.

Electrochemical sensors are highly sensitive, but the background signal is significantly affected by the electrode interface condition and solution components. Measurement stability is generally ensured by the measurement solvent and electrode polishing. If the measurement solvent contains foreign substances, a calibration curve must be prepared using the same solvent to maintain quantitation accuracy. In this study, we used a gold nanoparticle-modified boron-doped diamond electrode (AuNP-BDD) to measure the environmental standard of mercury without preparing a calibration curve for each soil elution sample (Figure 1). The time required to concentrate a certain amount of mercury in solution on electrodes while being stirred at a constant rate was used as an indicator of the presence of mercury.

## 2. Materials and Methods

### 2.1. Materials and Instrumentation

Mercury, lead, copper, and zinc standard solutions; a trivalent arsenic solution (arsenic (III)); acetic acid; and sodium acetate trihydrate were purchased from FUJIFILM Wako Pure Chemical Corporation (Osaka, Japan). An Ag/AgCl electrode was used as the reference electrode for the electrochemical measurements (BAS Inc., Tokyo, Japan). Electrochemical measurements were performed using an electrochemical analyzer (ALS 832D, BAS Inc., Tokyo, Japan). SEM analysis was conducted using JSM-9100F (JEOL Ltd., Tokyo, Japan) and Ultra Plus (Carl Zeiss Microscopy GmbH, Jena, Germany) instruments.

### 2.2. Time Response Electrochemical Detection

Electrochemical measurements were obtained using chemically synthesized and physically stable boron-doped diamond electrodes (BDDs). Gold nanoparticles (AuNPs) were modified on the surface to improve reactivity to mercury. BDDs were synthesized using chemical vapor deposition. An AuNP-BDD was prepared according to a previously reported electrochemical deposition method [26]. The gold nanoparticles were densely packed on a thick BDD film (Figure S1) using a high reduction voltage [27]. All electrochemical measurements were conducted in a 0.1 M Acetic acid/sodium acetate buffer (AcONa; pH 5, 20 °C). The measurements were performed using a three-electrode system with the AuNP-BDD, BDD, and Ag/AgCl as the working, counter, and reference electrodes, respectively. Square-wave anodic stripping voltammetry was used to measure the time response of the mercury detection method. Mercury and electrochemical stirring cells were added to a solution containing 0.1 M AcONa. The solution was stirred at 1000 rpm under an electrodeposition voltage of −0.7 V. Initially, the electrode surface was cleaned by applying a voltage of 0.7 V for 15 s.

### 2.3. Data Analysis of the Mercury Determination in Solution

Numerical processing was performed to minimize the differences in the background signals due to the electrode and solvent conditions. Three numerical value ranges were set as criteria for judgment. The second derivatives of the output data of the time resolution measurements were analyzed in the range of 0.608–0.8 V. Mercury above a specified concentration was detected when the graph peak intensity of the output second-order derivative was between −0.001 and −0.003, the width at half maximum was between 0.99 and 0.2, and the peak position was 0.68 ± 0.05 V. The parameters for mercury concentration discrimination were determined from random electrochemical measurements for all electrodeposition times. All measurement solvents were mercury-free (Table S1).

### 2.4. Electrochemical Detection of Mercury in a Soil Eluent

The soil eluent was prepared by adding 50 mL of a buffer solution to 5 g of soil and shaking for 1 min. After shaking, the solution was filtered to remove the soil, and only the solution component was used for the measurements. The absorbances of the eluent and buffer were measured to determine the presence of proteins. The presence of mercury in the actual specimen was determined by adding mercury to the soil eluent and measuring electrochemically using the same procedure.

## 3. Results and Discussion

### 3.1. Property of a Gold Nanoparticle-Modified Boron-Doped Diamond Electrode

**Figure 2:**
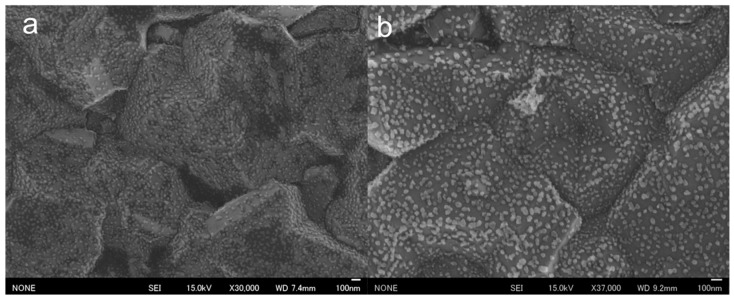
(**a**) SEM observations of the surface of a gold nanoparticle-modified boron-doped diamond electrode (AuNP-BDD). (**b**) SEM observations of the surface of an AuNP-BDD after 50 CV cycles.

SEM observations of the surfaces of the prepared AuNP-BDDs showed that the nanoparticles were tightly arranged on micro-sized irregularities. The BDDs were fabricated under polycrystalline conditions with a film thickness of approximately 200 µm, and the surface irregularities were indicative of polycrystalline diamond properties (Figure 2a). Gold nanoparticles were formed by the reduction of gold ions on the electrode surface of a highly concentrated solution, suggesting that the electrochemical reduction of gold ions was successfully completed, resulting in the formation of numerous nanomaterials on relatively uniform grains on the BDDs. In a conventional electrochemical modification process, gold ions are typically subjected to low-voltage conditions ranging between −0.4 and −0.7 V [28]. However, when using BDD electrodes, electrolysis of water occurs at the higher negative voltages. This unique phenomenon enables the dense modification of nanoparticles within a short timeframe at high voltages. To evaluate the electrochemical stability of the fabricated AuNP-BDD, cyclic voltammetry (CV) was conducted for 50 cycles in the voltage range used for the mercury detection experiments (Figure S1A). A characteristic current peak appeared at approximately 0.3–0.4 V when gold nanoparticles were used as the working electrode. This peak was observed in both positive and negative sweep directions, which is characteristic of using gold material as the working electrode. Although a cycle-dependent decrease in the characteristic peak was observed, no peak disappearance occurred, suggesting that the gold nanoparticles on the electrode surface were not significantly desorbed or ionized, even with prolonged voltage application. After 50 cycles, SEM analysis confirmed that the electrode surface remained modified with a large number of gold nanoparticles (Figure 2b). This result validates the electrochemical stability observed from CV. Ionization due to oxidation of the gold material occurred over time with the application of voltages above 0.8 V. In the mercury detection technique proposed in this study, the voltage was swept up to 0.8 V, which did not significantly affect repeated measurements using the same electrode. To evaluate the performance of the AuNP-BDD as an electrode, impedance measurements were performed, revealing that the electrical resistance of the electrode was significantly reduced by nanoparticle modification compared to that of the BDD. The interactions between the nanoparticles on the surface of the AuNP-BDD enhanced the efficient transfer of electrons at the electrode interface. Furthermore, the impedance of the AuNP-BDD was measured after 50 CV cycles (Figure S1B). The electrical resistance remained relatively unchanged before and after the tests, demonstrating that the AuNP-BDD showed excellent properties as a highly active electrode for mercury detection.

### 3.2. Optimization of the Measurement Conditions for High-Sensitivity Mercury Detection

**Figure 3:**
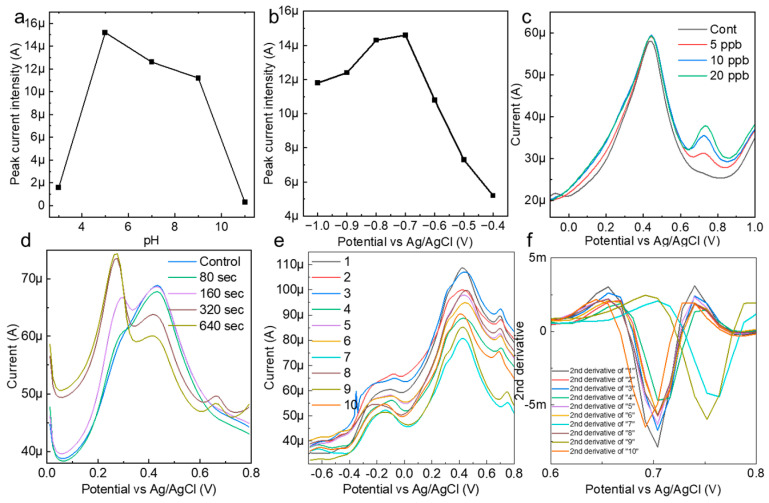
(**a**) Confirmation of the pH dependence of the peak current value during the mercury detection measurements. (**b**) Confirmation of the electrodeposition voltage and peak current intensity for the optimization of the mercury detection conditions. (**c**) A mercury detection test in AcONa under the optimal electrochemical measurement conditions. (**d**) The electrodeposition time and peak current intensity for the detection of 0.5 ppb mercury in AcONa. (**e**) Electrochemical measurements of 10 ppb mercury in AcONa using a random state AuNP-BDD to clarify background signals due to individual electrode differences and attrition rates. (**f**) Spectrum obtained by second-order differentiation of measured current values in the voltage range from 0.608 to 0.8 V.

The reactivity of the AuNP-BDD with mercury ions in the solution was highly dependent on the pH of the solution. To establish optimal measurement conditions, the pH of AcONa was varied between 3 and 11, and mercury detection at 10 ppb was performed (Figure 3a). Details of the pH preparation of AcONa are provided in Supplementary Material. The results show that the peak current increase caused by mercury ionization was greatest at pH 5 and that the peak current values were relatively stable up to pH 9. This indicates that mercury ions were most efficiently concentrated on the electrode under weakly acidic conditions. Based on this result, a solution of pH 5 was used as the best solvent for subsequent tests. The electrochemical measurement of mercury was also stable when the actual sample did not contain soil that was strongly alkaline or acidic.

To optimize the measurement of mercury, the relationship between the electrodeposition voltage and peak current was evaluated, and the strongest mercury-peak current was observed under an applied voltage condition of −0.7 V in AcONa (Figure 3b). Mercury measurements in the range of 5–20 ppb were performed under optimal pH and electrodeposition voltage conditions (Figure 3c). A characteristic mercury-peak current appeared at approximately 0.68, suggesting that the AuNP-BDD was a highly active electrode that was capable of measuring low concentrations of mercury at the ppb level. To further evaluate the sensitivity of the AuNP-BDD electrode for mercury detection, the dependence of the peak current intensity on the electrodeposition time in a low-concentration (0.5 ppb) mercury solution was confirmed (Figure 3d). A small mercury reaction was observed after 160 s. Notably, no intensity differences in the peak current values were observed beyond electrodeposition times of 320 and 640 s, suggesting that the reaction reached its maximum extent at a mercury concentration of 0.5 ppb. On the other hand, the intensity of the peak at approximately 0.2 V increased with electrodeposition time, suggesting that silver ions leached from the reference electrode were deposited on the AuNP-BDD.

AuNP-BDDs were randomly immersed in a 10 ppb mercury solution, including those using different solutions immediately after synthesis under the same conditions up to the reuse of the electrodes, and the signal differences were compared during the electrodeposition process for 300 s (Figure 3e). The graph shows that there is little difference in the height of the peak intensity between the electrodes, whereas there is a large difference in the value of the background signal. In the case of nanoparticle-modified electrodes, particle depletion owing to the application of a voltage to the surface particles and density differences during modification appeared as individual differences. When mechanical discrimination was performed based on current values alone, these differences in background signals owing to individual differences between electrodes had a significant impact on the optimal discrimination result output. To address this point, a peak analysis was performed using the second-order derivative and graph maximum as the baseline in the range of 0.608–0.8 V among the differentiated values (Figure 3f). Graphing the numerical values output by the second-order derivative showed the percentage change in the rate of change, which did not reflect the intensity difference caused by the background signal. This made it possible to discriminate the method proposed in this study from a mechanical process that suppressed the effects of individual electrode differences, even if the analysis was only based on the intensity from a simple baseline. In this study, we aimed to improve the accuracy by using the three parameters of the output peak potential, intensity from the baseline, and peak half-width for discrimination. In electrochemistry, contamination of the reference electrode and rapid changes in the solvent pH often result in incorrect control of the applied voltage. The use of multiple parameters enabled error avoidance.

### 3.3. Time Response Detection of Low-Concentration Mercury

**Figure 4:**
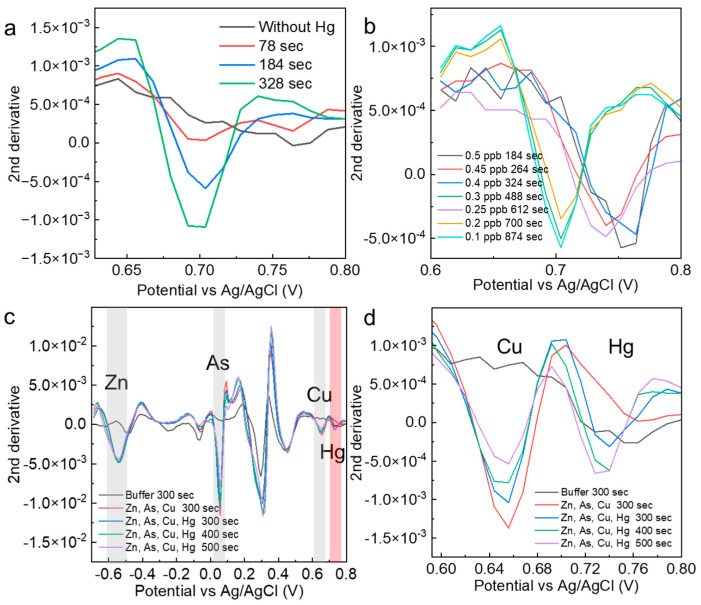
(**a**) Second derivative spectra analyzed from measurements of 0.5 ppb mercury at different electrodeposition times. (**b**) Optimization results of the mercury electrodeposition time for the determination of specific mercury concentrations. (**c**,**d**) Confirmation of the mercury selectivity of mercury measurements in solutions containing other heavy metals. The red shade indicates Hg, and the gray shade indicates the electrochemical peaks of other heavy metals.

To demonstrate that the electrodeposition time is effective in determining the presence of specific concentrations of mercury, mercury (0.5 ppb) was measured at different electrodeposition times. The results show that the peak intensity at 78 s was very small and did not meet the criterion of low variation obtained from the control measurement. At 184 s, a value that fully satisfied the criterion was obtained, and at 324 s, a peak value with an even higher intensity was obtained. The optimal number of electrodeposition seconds is essential for determining specific concentrations of mercury. The electrodeposition time for very low concentrations of mercury (0.1–0.5 ppb) was minimized in an experimental system in which the solution agitation speed was increased from 1000 rpm when the conditions were examined (Figure 4b). The heavy metals added here are those commonly identified in various soil distribution cases. As a result, the optimum electrodeposition time for each low mercury concentration was stable. Although the probability of contact with the electrode depended on the stirring speed, stirring time, and structure of the measurement site, we believed that once the optimum time was set in an established experimental system, stable determination of the concentration would be possible. The mercury-peak current values were evaluated when other foreign heavy metal ions were added to 0.5 and 10 ppb mercury solutions (Figure 4c). For zinc, the peak intensity was not dependent on the electrodeposition time, suggesting that the entire amount was quickly deposited on the electrode in the solution. For arsenic and copper, time dependence was observed. In the solvent used in this study, copper ions showed current peaks similar to those of mercury (Figure 4d). The mercury peak showed a sufficient intensity at 300 s, even when the peak current of copper occurred in the vicinity of the mercury peak. In the AcONa buffer, the mercury concentration was determined with high selectivity and sensitivity. This measurement method based on the electrodeposition time was applied to other heavy metals, and the time dependence of the current values was evaluated for arsenic and lead at a concentration of 10 ppb. For both ions, clear peaks at electrodeposition times of less than 15 s were confirmed. Combined with the analysis, these results suggest that low concentrations could be measured without the need for electrodeposition (Figure S2).

### 3.4. Real Sample Measurement

**Figure 5:**
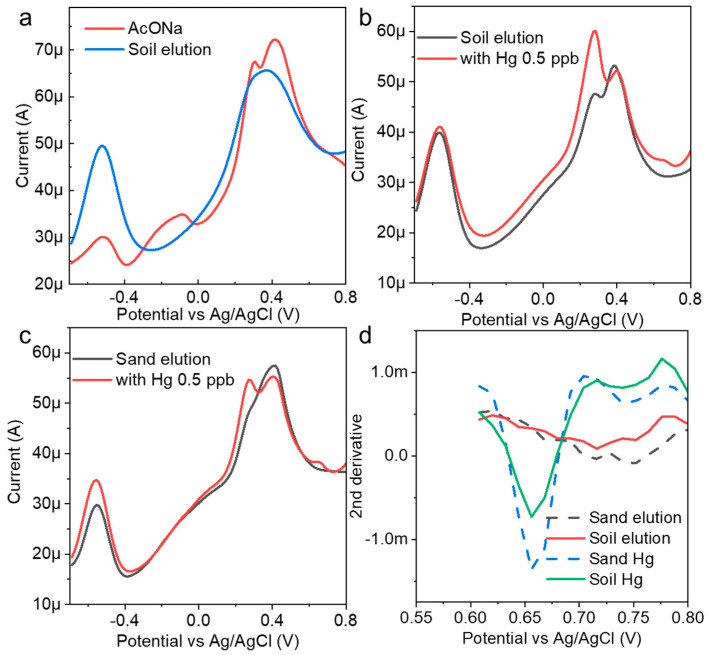
(**a**) Electrochemical measurement of an AuNP-BDD in a mixture of AcONA and a soil eluent; mercury detection results (0.5 ppb) in (**b**) soil and (**c**) sand eluents. (**d**) Results of the mercury concentration determination using analytical methods.

Soil eluents were prepared with AcONa using simple filtering and were used as spike samples. Electrochemical measurements were performed without mercury, and a large current peak at approximately −0.5 V was obtained (Figure 5a). In this region, a zinc oxidation voltage was often observed. It was suggested that, at least in the eluent, heavy metals other than mercury were electrodeposited on the electrode surface, and a strong current peak was confirmed. When mercury (0.5 ppb) was added to the eluent prepared from the soil samples and measured to determine the concentration, the peak current of mercury appeared at approximately 0.68 V (Figure 5b). This suggested that the AuNP-BDD could detect low concentrations of mercury in eluents. The eluent was prepared under similar conditions using commercial sand as a sample with different properties (Figure 5c). The results of the electrochemical measurements before and after the addition of mercury indicate that the peak current value presumed to be zinc was lower in intensity than that of the soil specimen. The obtained mercury-peak current was stronger, suggesting that the presence of foreign substances affected the intensity of the peak current. The eluent contained a large qualitative and quantitative difference in contaminants in each soil used, and calibration curve preparation for electrochemical quantification required the use of a sample eluent for electrochemical quantification with only simple filtering. When the results of both measurements were submitted for analysis, it was determined that 0.5 ppb mercury was eluted in both cases (Figure 5d). This result suggests that concentration determination based on the optimum electrodeposition time could be combined with analysis to determine the mercury concentration from soil samples with different properties. For example, the hydrochloric acid elution method efficiently elutes mercury and allows for its quantification through the luminescence method following chemical treatment. Notably, this approach yields a highly reliable calibration curve, even within the very low concentration range of 19 ng/g [29]. In contrast, the present study demonstrates the advantage of electrochemical determination in detecting very low concentrations of inorganic mercury with minimal pretreatment.

## 4. Conclusions

In this study, an electrochemical mercury concentration discrimination method was devised. This method was based on the analysis of current peaks obtained from electrodeposition times under specific electrode and solvent conditions. It was characterized by its low error determination under low concentration conditions and detected mercury at 0.5 ppb in a soil leachate and dilution series of standard reagents. Thus, an electrochemical sensor that was less susceptible to significant sensitivity loss even in the presence of foreign substances was realized. However, there remain issues that need to be addressed with regard to the extraction stage that realizes a simple on-site test for mercury. This research will allow the determination of low concentrations of mercury in solutions containing many residual micro-compounds in soil without the need for calibration curves.

## Data Availability

Data are contained within the article and Supplementary Materials.

## Supplementary Materials

The following supporting information can be downloaded at https://www.mdpi.com/article/10.3390/nano14110981/s1. Figure S1: Confirmation of the time-dependent peak current value changes with square-wave stripping voltammetry for arsenic and lead; Figure S2. Electrochemical cyclic voltammetry (CV) measurements using an AuNP-BDD as the working electrode stripping voltanmetry for As and Pb; Table S1: Random electrochemical control measurements.

## References

[1] Clarkson T.W. (1997). The toxicology of mercury. Crit Rev. Clin. Lab Sci..

[2] Rice K.M., Walker E.M., Wu M., Gillette C., Blough E.R. (2014). Environmental mercury and its toxic effects. J. Prev. Med. Public Health.

[3] Driscoll C.T., Mason R.P., Chan H.M., Jacob D.J., Pirrone N. (2013). Mercury as a global pollutant: Sources, pathways, and effects. Environ. Sci. Technol..

[4] Boening D.W. (2000). Ecological effects, transport, and fate of mercury: A general review. Chemosphere.

[5] Sakata M., Marumoto K. (2005). Wet and dry deposition fluxes of mercury in Japan. Atmos. Environ..

[6] Zhang Y., Nakai S., Masunaga S. (2009). An exposure assessment of methyl mercury via fish consumption for the Japanese population. Risk Anal..

[7] Li P., Feng X.B., Qiu G.L., Shang L.H., Li Z.G. (2009). Mercury pollution in Asia: A review of the contaminated sites. J. Hazard. Mater..

[8] Basu N., Horvat M., Evers D.C., Zastenskaya I., Weihe P., Tempowski J. (2018). A state-of-the-science review of mercury biomarkers in human populations worldwide between 2000 and 2018. Environ. Health Perspect..

[9] dos Santos J.S., de la Guárdia M., Pastor A., dos Santos M.L.P. (2009). Determination of organic and inorganic mercury species in water and sediment samples by HPLC on-line coupled with ICP-MS. Talanta.

[10] Li Y., Chen C., Li B., Sun J., Wang J., Gao Y., Zhao Y., Chai Z. (2006). Elimination efficiency of different reagents for the memory effect of mercury using ICP-MS. J. Anal. At. Spectrom..

[11] Jagtap R., Maher W. (2015). Measurement of mercury species in sediments and soils by HPLC–ICPMS. Microchem. J..

[12] Hylander L.D., Goodsite M.E. (2006). Environmental costs of mercury pollution. Sci. Total. Environ..

[13] Feng X.-B., Chou A.-L., Fu H.-W., He T.-R., Li B., Yu S.-F. (2009). Mercury pollution in the environment. Prog. Chem..

[14] Budnik L.T., Casteleyn L. (2019). Mercury pollution in modern times and its socio-medical consequences. Sci. Total. Environ..

[15] Gustin M.S., Evers D.C., Bank M.S., Hammerschmidt C.R., Pierce A., Basu N., Blum J., Bustamante P., Chen C., Driscoll C.T. (2016). Importance of Integration and Implementation of Emerging and Future Mercury Research Into the Minamata Convention. Environ. Sci. Technol..

[16] Huang J.-H., Shetaya W.H., Osterwalder S. (2020). Determination of (Bio)-available mercury in soils: A review. Environ. Pollut..

[17] Kumari S., Amit R., Jamwal R., Mishra N., Singh D.K. (2020). Recent developments in environmental mercury bioremediation and its toxicity: A review. Environ. Nanotechnol. Monit. Manag..

[18] Cabañero Ortiz A.I., Madrid Albarrán Y., Cámara Rica C. (2002). Evaluation of different sample pre-treatment and extraction procedures for mercury speciation in fish samples. J. Anal. At. Spectrom..

[19] Qvarnström J., Frech W. (2002). Mercury species transformations during sample pre-treatment of biological tissues studied by HPLC-ICP-MS. J. Anal. At. Spectrom..

[20] Leopold K., Foulkes M., Worsfold P. (2010). Methods for the determination and speciation of mercury in natural waters—A review. Anal. Chim. Acta..

[21] Miao P., Liu L., Li Y., Li G. (2009). A novel electrochemical method to detect mercury (II) ions. Electrochem. Commun..

[22] Du J., Jiang L., Shao Q., Liu X., Marks R.S., Ma J., Chen X. (2013). Colorimetric detection of mercury ions based on plasmonic nanoparticles. Small.

[23] Ye B.C., Yin B.C. (2008). Highly sensitive detection of mercury (II). Angew. Chem. Int. Ed. Engl..

[24] Balasurya S., Syed A., Thomas A.M., Marraiki N., Elgorban A.M., Raju L.L., Das A., Khan S.S. (2020). Rapid colorimetric detection of mercury using silver nanoparticles in the presence of methionine. Spectrochim. Acta A Mol. Biomol. Spectrosc..

[25] Horvat M. (2005). Determination of mercury and its compounds in water, sediment, soil and biological samples. Dynamics of Mercury Pollution on Regional and Global Scales: Atmospheric Processes and Human Exposures Around the World.

[26] Ohmagari S., Srimongkon K., Yamada H., Umezawa H., Tsubouchi N., Chayahara A., Shikata S., Mokuno Y. (2015). Low resistivity p+ diamond (100) films fabricated by hot-filament chemical vapor deposition. Diam. Relat. Mater..

[27] Takemura K., Iwasaki W., Morita N., Ohmagari S. (2022). High density and monodisperse electrochemical gold nanoparticle synthesis utilizing the properties of boron-doped diamond electrodes. Nanomaterials.

[28] Bottari F., De Wael K. (2017). Electrodeposition of gold nanoparticles on boron doped diamond electrodes for the enhanced reduction of small organic molecules. J. Electroanal. Chem..

[29] Kodamatani H., Tomiyasu T. (2013). Selective determination method for measurement of methylmercury and ethylmercury in soil/sediment samples using high-performance liquid chromatography–chemiluminescence detection coupled with simple extraction technique. J. Chromatogr. A.

