# Supplementary material for: Determination of Low Concentrations of Mercury Based on the Electrodeposition Time
Source: Nanomaterials (Basel). 2024 Jun 5;14(11):981. doi: 10.3390/nano14110981 (PMC11173952; doi:10.3390/nano14110981)
Supplement: Supplementary file 1 [file nanomaterials-14-00981-s001.zip › nanomaterials-3031825-supplementary.pdf]

Supporting information

Determination of Low Concentrations of Mercury Based on the Electrodeposition Time

Kenshin Takemura [1,*], Wataru Iwasaki [1], Nobutomo Morita [1], Shinya Ohmagari [1], Yasunori Takaki [2], Hitomi Fukaura [2] and Kazuya Kikunaga [1]

[1] Sensing System Research Center, National Institute of Advanced Industrial Science and Technology (AIST), 807-1 Shuku-Machi, Tosu 841-0052, Saga, Japan; wataru.iwasaki@aist.go.jp (W.I.); morita.nobutomo@aist.go.jp (N.M.); shinya.ohmagari@aist.go.jp (S.O.); k-kikunaga@aist.go.jp (K.K.)

[2] Sakamoto Lime Industry Co., Ltd., 273-1 Simo, Tamana 865-0013, Kumamoto, Japan; k-kaihatu01@sakamoto-lime.com (Y.T.); k-kaihatu02@sakamoto-lime.com (H.F.)

* Correspondence: takemura.kenshin@aist.go.jp

Keywords: time response; nanoparticle; electrochemical detection; mercury; environment

Table S1. Random control measurements

Sample No.	Deposition time (sec)	Peak intensity	Peak potential	FWHM
1	1000	-7.84E-04	0.70134	0.128
2	100	-7.76E-04	0.75669	0.114
3	1000	-6.02E-04	0.69786	0.03432
4	1000	-4.77E-04	0.6974	0.1554
5	500	-5.99E-04	0.70418	0.1172
6	100	-5.16E-04	0.72303	0.1138
7	200	-6.46E-04	0.75508	0.11823
8	900	-0.00279	0.68475	0.03675
9	500	-5.99E-04	0.70418	0.03891
10	500	-0.00162	0.74584	0.10149
11	600	-4.83E-04	0.67429	0.0435
12	680	-8.24E-04	0.6923	0.0638
13	200	-0.00113	0.75685	-8.67E-04
14	300	-0.00111	0.78264	0.12882
15	264	-0.00108	0.75598	0.10959
16	264	-8.59E-04	0.76209	0.12668
17	264	-3.01E-04	0.71184	0.02064
18	264	-6.41E-04	0.76343	0.11391
19	264	-7.62E-04	0.76681	0.1159
20	288	-0.00114	0.76676	0.11486
21	300	-3.40E-04	0.70644	0.03682
22	318	-5.93E-04	0.75599	0.06392
23	312	-2.45E-04	0.65513	0.06903
24	324	-6.03E-04	0.74851	0.06531
25	300	-0.00102	0.7716	0.0501
26	324	-9.98E-04	0.70513	0.0245
27	330	-9.34E-04	0.76061	0.11185
28	336	-7.71E-04	0.76076	0.13341
29	300	-9.09E-04	0.68425	0.0738

30	336	-6.10E-04	0.68442	0.05843
31	300	-5.59E-04	0.7123	0.03294
32	336	-4.44E-04	0.70883	0.02497
33	312	-3.42E-04	0.70289	0.02105
34	336	-3.21E-04	0.7103	0.00992
35	336	-7.26E-04	0.71882	0.09852
36	336	-5.90E-04	0.67195	0.08762
37	336	-6.37E-04	0.75974	0.09707
38	300	-2.65E-04	0.71575	0.02396
39	300	-4.97E-04	0.6915	0.04299
40	300	-7.11E-04	0.70121	0.05251
41	544	-9.40E-04	0.74375	0.12924
42	544	-5.03E-04	0.75833	0.07667
43	544	-4.25E-04	0.761	0.04429
44	544	-3.69E-04	0.75666	0.04419
45	300	-2.62E-04	0.7045	0.03198
46	80	-3.68E-04	0.71954	0.11524
47	84	-6.49E-04	0.74196	0.12685
48	88	-6.87E-04	0.74285	0.11685
49	96	-7.24E-04	0.75854	0.13776
50	104	-6.25E-04	0.74006	0.10254
51	300	-8.83E-04	0.67278	0.04468
52	544	-8.37E-04	0.70093	0.06417
53	544	-8.41E-04	0.80136	0.23873
54	300	-5.60E-04	0.65744	0.04035
55	544	-4.16E-04	0.67782	0.05246
56	680	-6.36E-04	0.69362	0.04824
57	72	-9.54E-04	0.75644	0.13978
58	76	-5.90E-04	0.75921	0.12695
59	80	-3.78E-04	0.73692	0.06901
60	80	-6.50E-04	0.76557	0.1324
61	900	-9.25E-04	0.8277	0.2615
62	300	-0.00221	0.67356	0.3036
63	224	-0.00105	0.76787	0.11659
64	252	-8.49E-04	0.7602	0.11244
65	280	-7.56E-04	0.7531	0.10284

66	224	-7.60E-04	0.74752	0.09487
67	900	-7.53E-04	0.69747	0.04172
68	500	-0.00782	0.66563	0.07226
69	1600	NA	NA	NA
70	300	NA	NA	NA
71	544	NA	NA	NA
72	300	NA	NA	NA
73	300	NA	NA	NA
74	600	NA	NA	NA

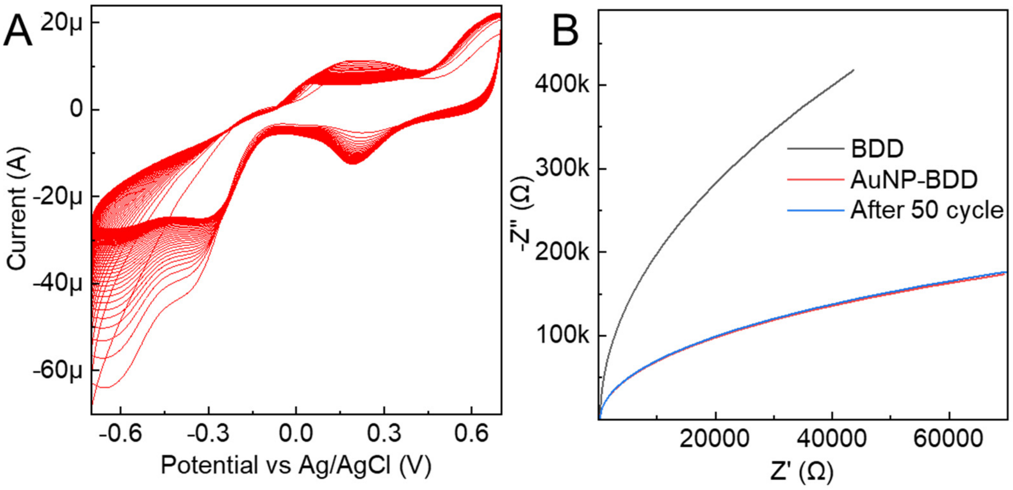

Figure S1. (**a**) Electrochemical cyclic voltammetry (CV) measurements using an AuNP-BDD as the working electrode; (**b**) Impedance measurement results using various electrodes. The black and red lines correspond to the BDD and AuNP-BDD, respectively. Furthermore, the blue line corresponds to a Cole–Cole plot of an AuNP-BDD after 50 CV cycles.

Supporting experimental procedure for pH adjustment of AcONa

AcONA buffer was prepared by mixing 0.1 M acetic acid and 0.1 M sodium acetate at a fixed ratio. A pH of 4 was prepared by mixing 164 ml of acetic acid and 36 ml of sodium acetate. A pH of 5 was prepared by mixing 59 ml of acetic acid and 141 ml of sodium acetate. The range of buffers from pH 6 onward that showed no buffering capacity was prepared by adding a 0.1 M sodium hydroxide solution to AcONa prepared at pH 5.6. pH 3 solutions were prepared by adding 0.1 M nitric acid to AcONa prepared at pH 3.6.

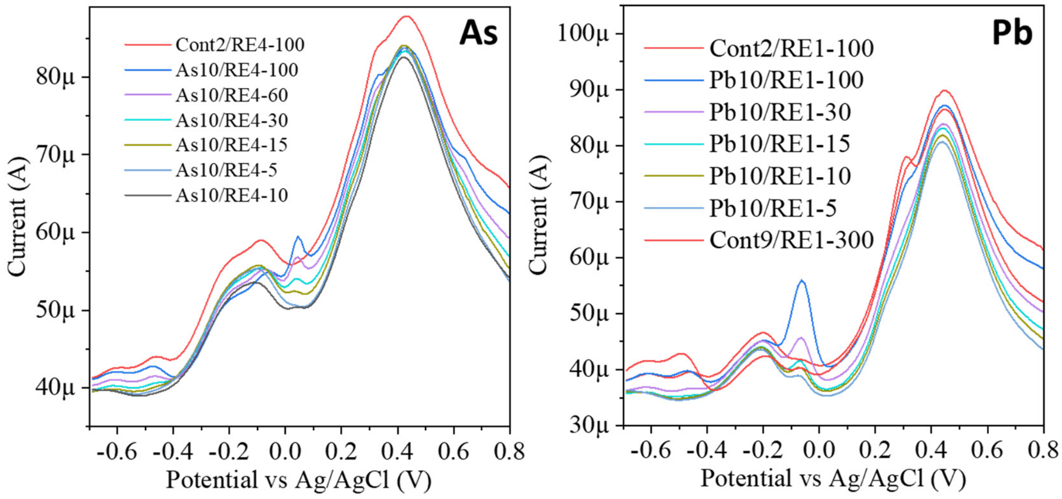

Figure S2. Electrochemical cyclic voltammetry (CV) measurements using an AuNP-BDD as the working electrode stripping voltanmetry for As and Pb.
